# Latest Innovations and Nanotechnologies with Curcumin as a Nature-Inspired Photosensitizer Applied in the Photodynamic Therapy of Cancer

**DOI:** 10.3390/pharmaceutics13101562

**Published:** 2021-09-26

**Authors:** Laura Marinela Ailioaie, Constantin Ailioaie, Gerhard Litscher

**Affiliations:** 1 Department of Medical Physics, Alexandru Ioan Cuza University, 11 Carol I Boulevard, 700506 Iasi, Romania; lauraailioaie@yahoo.com (L.M.A.); laserail_mail@yahoo.com (C.A.); 2 President of ISLA (International Society for Medical Laser Applications), Research Unit of Biomedical Engineering in Anesthesia and Intensive Care Medicine, Research Unit for Complementary and Integrative Laser Medicine, and Traditional Chinese Medicine (TCM) Research Center Graz, Medical University of Graz, Auenbruggerplatz 39, 8036 Graz, Austria

**Keywords:** light, malignant tumors, nanomedicine, natural photosensitizer, photobiomodulation (PBM), photodynamic therapy (PDT), cancer, curcumin

## Abstract

In the context of the high incidence of cancer worldwide, state-of-the-art photodynamic therapy (PDT) has entered as a usual protocol of attempting to eradicate cancer as a minimally invasive procedure, along with pharmacological resources and radiation therapy. The photosensitizer (PS) excited at certain wavelengths of the applied light source, in the presence of oxygen releases several free radicals and various oxidation products with high cytotoxic potential, which will lead to cell death in irradiated cancerous tissues. Current research focuses on the potential of natural products as a superior generation of photosensitizers, which through the latest nanotechnologies target tumors better, are less toxic to neighboring tissues, but at the same time, have improved light absorption for the more aggressive and widespread forms of cancer. Curcumin incorporated into nanotechnologies has a higher intracellular absorption, a higher targeting rate, increased toxicity to tumor cells, accelerates the activity of caspases and DNA cleavage, decreases the mitochondrial activity of cancer cells, decreases their viability and proliferation, decreases angiogenesis, and finally induces apoptosis. It reduces the size of the primary tumor, reverses multidrug resistance in chemotherapy and decreases resistance to radiation therapy in neoplasms. Current research has shown that the use of PDT and nanoformulations of curcumin has a modulating effect on ROS generation, so light or laser irradiation will lead to excessive ROS growth, while nanocurcumin will reduce the activation of ROS-producing enzymes or will determine the quick removal of ROS, seemingly opposite but synergistic phenomena by inducing neoplasm apoptosis, but at the same time, accelerating the repair of nearby tissue. The latest curcumin nanoformulations have a huge potential to optimize PDT, to overcome major side effects, resistance to chemotherapy, relapses and metastases. All the studies reviewed and presented revealed great potential for the applicability of nanoformulations of curcumin and PDT in cancer therapy.

## 1. Introduction

Light as a treatment dates back to ancient times, but modern photodynamic therapy (PDT) has advanced following the accidental rediscovery in the early twentieth century of the light-mediated killing effect in the presence of molecular oxygen on acridine-incubated *Paramecium caudatum* [1].

Exactly in the same direction, concerning the use of light in medicine, the first Nobel Prize for outstanding applications of phototherapy was won in 1903 by Niels Finsen for smallpox and skin tuberculosis treatments [2,3].

Herman von Tappeiner defined “photodynamic action” [4] and gave it the name known today as photodynamic therapy, a contemporary and non-invasive form of anticancer therapy, well studied in present also for infections, non-oncological disorders, and in some countries, as a standardized protocol, along with radiotherapy and chemotherapy in anti-cancer treatments [5,6].

The first goal of this review was to present and discuss the latest nanotechnologies in relation to curcumin as photosensitizer (PS) to optimize photodynamic therapy (PDT), to overcome major side effects, resistance to chemotherapy, relapses and metastases.

The second objective was to reveal the state of the art of photodynamic therapy as a field of continuous effervescent research and medical applications in direct connection with nanoformulations of curcumin.

The third aim was to analyze the molecular and cellular mechanisms, targeted delivery and localization of nanoparticles in the tumor, as well as the interconnected parts related to PDT, to increase the antiproliferative and apoptotic activity and minimize toxicity in nearby healthy cells.

The fourth goal was to push forward the field of study of natural photosensitizers, especially curcumin- and PDT-related nanotechnologies, providing an up-to-date high-quality evidence base for researchers and scientists to accelerate and rethink new experiments and innovations in cancer.

## 2. Photosensitizers and Photodynamic Therapy

Photosensitizers (PSs) are molecules unchanged before and after energy exchange, that can absorb electromagnetic radiation from infra-red, visible and UV range and trigger the physicochemical change of a neighboring molecule by yielding an electron to the substrate or by extracting an atom from it, and finally, the PS returns to its ground state, where it remains unchanged until it absorbs radiation again [6,7].

**Figure 1:**
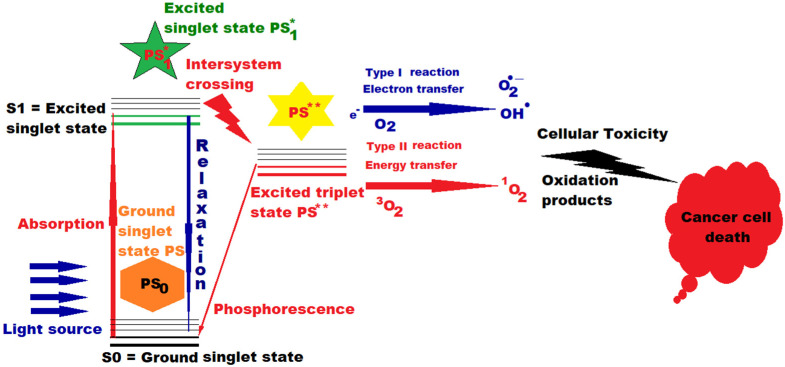
Action of PDT and photosensitizer (PS) in cancer therapy. A single asterisk “*” means “excited singlet state”, and two asterisks “**” mean “excited triplet state”.

When incident photons are absorbed, PS advances an electron in a single excited state, which, through an intrinsic spin rotation, can pass into an excited triplet state with a longer lifetime, thus increasing the probability of PS interaction with next-door molecules, and finally, conducting to the selective death of diseased cells through the generation of cytotoxic oxidation species (Figure 1). Depending on the internal structure of the PS, they have different efficiencies when interacting with diverse wavelengths [6,8].

PS had an inherent basis in nature through the green pigment chlorophyll, existing in all green plants and in cyanobacteria, absorbing light to deliver the photosynthesis’ energy, as well as other light-sensitive molecules in the plant kingdom.

PDT implies the selective sensitization of tissues to light and the first studies on PS started at the beginning of the last century, when researchers noticed this phenomenon and undertook investigation of it in malignant tumors [9].

In the middle of the last century, Figge et al. showed that exogenous porphyrins accumulated selectively in murine tumors [10] and the results were afterwards applied to cancer patients by first injecting raw hematoporphyrins [11], followed by improvement using a hematoporphyrin “derivative” which highlighted the enhanced selective fluorescence of the neoplasms [12,13].

However, we could consider that the contemporary version of PDT was born in the 1970s in the United States due to the activity of Dr. T.J. Dougherty and collaborators, who later used the more refined version of the “hematoporphyrin derivative”, called Photofrin, the most implemented PS worldwide, even nowadays, despite the many difficulties (long-term photosensitivity of the skin of cancer patients treated, reduced absorption in large tumors, due to the limited penetration of photons etc.) [14].

In a paper published 25 years later by Thomas J. Dougherty et al., PDT is well defined as involving the management of a PS, which may also need a metabolic combination (a pro-drug), followed by exposure to light with a certain wavelength for maximum absorption. The effect is an irreversible succession of photochemical and photobiological reactions with the permanent photodeterioration of malignant cells. Preclinical and clinical research has established PDT as a useful care procedure in early or advanced stages for lung, digestive, genitourinary cancer, etc. [15].

The merit of Thomas Dougherty’s pre-clinical and clinical trials and efforts, led to FDA approval of PDT in modern clinical practice, and paved the way for current and future advances in PDT, coupled nowadays with nanotechnologies and subsequent drug discoveries [16].

Without Dougherty’s endeavor, it is questionable that PDT would not have remained just “a minor biomedical curiosity” [17].

PDT is a therapy that comprises light, a chemical compound that makes cells abnormally sensitive or reactive to light, and in the presence of tissue oxygen induces cell death by generated reactive oxygen species (ROS), i.e., through phototoxicity. Today, this technology is extensively used for certain conditions, incorporating the latest applications in antiviral treatments and cancers that tend to metastasize. For example, in practically applied PDT, the light with specific wavelengths is usually guided by optical fibers to the patient’s tumor, which has been given a photoactive drug, which will attach intensely to abnormal cells. The light will stimulate the substance used for treatment, will generate cytotoxic species in the presence of oxygen, and thus, the malignant cells could be destroyed, procedure considered to be invasively negligible and, to a lesser extent, toxic. A disadvantage is the long-term photosensitization, unpleasant and annoying for patients, but counterbalanced by the diminished necessity for fine operations, shortening recovery time and reducing cicatrices or deformities to the smallest possible size [6,18].

PDT practice implies: the PS, the light irradiation system (L) and the molecular oxygen of the tissue (O_2_). Wavelength of L must be quantum suited to move PS into action to generate free radicals (type I reaction), as a result of electronic extraction or relocation to an underlying molecule and/or reactive oxygen species, especially singlet oxygen (type II reaction), an extremely reactive species. PDT is composed of several consecutive phases. First and foremost, PS is applied without light, either systemically (intravenous administration), or by topical application. Thereafter, when enough PS is fused into the unhealthy cells to be destroyed, the PS is stimulated by setting the light for a well-defined time interval. The applied dose of electromagnetic radiation provides enough energy to advance PS to higher excitation energy states, but not sufficient to deteriorate the adjacent healthy cells. In the next step of relaxation are generated reactive oxygen species that will put to death the targeted tissue. Unlike other molecules that are normally in a singlet state, molecular oxygen in the atmosphere and in living cells exists in a triplet state, but because quantum physics interdicts reactions between triplets and singlets, this postulates molecular oxygen as inactive in normal states. However, the PS used in medical applications can undergo a process of intersection with oxygen during excitation, a process during which, from an excited singlet state, it will pass to an excited triplet state at the point where the two potential energy curves crosses, as presented in Figure 1 (intersystem crossing), giving rise also to phosphorescence, and consequently, the extremely cytotoxic singlet oxygen will be generated, which will attack any organic substance with which it comes into contact, being meanwhile eliminated very quickly in less than 3 microseconds from the illuminated cells [7,19,20].

When excited in type II reactions, PS provides its excess energy during the process of interacting with molecular triplet oxygen (^3^O_2_) and produces singlet oxygen (^1^O_2_), a highly reactive species that reacts with the substrate to generate other oxidized products that will attack cellular constituents, leading to the targeted killing of cells in the supplied light field.

PSs contain chromophores, i.e., they are photosensitizers. A part of a molecule responsible for its color, is that part of the molecule in which the energy gap between two different molecular orbitals is in the visible area of the spectrum and, when it meets light and absorbs a photon, an electron from its ground state will pass into an excited state, and a conformational change of the molecule will occur. Once stimulated, the photosensitizer passes from the baseline S0 (ground state) into the short-lived excited state, with lots of vibrational sub-levels, it can decrease its energy by rapidly dropping these sub-levels via inner adjustment to populate the first excited singlet state S1, before it quickly relaxes back to S0 (see Figure 1). The transition from S1 to S0 is via fluorescence, with very short lifespans (10^−9^–10^−6^ s). From S1, it can pass through spin switch via intersystem crossing and can occupy the first excited triplet state, and after that to go downhill to baseline via phosphorescence, with a much longer lifespan (10^−3^ − 1 s); enough to enable the PS in excited triplet state to act on circumambient bio-compounds via type I and type II reactions [7,21].

Singlet oxygen produces effects at 10–55 nm from its origin in about 10–320 ns and can diffuse up to approximately 300 nm in vivo [7,21,22]. All time sequences involved in PS photoactivation and in type I and II reactions play a key role in disrupting the cellular machine, but type II reactions are deemed to be most efficient for cell impair, leading to the ultimate goal of killing unhealthy photo irradiated cells. However, in real practice, PSs with a triplet state life of less than 20 ns could still prove to be productive photodynamic agents [21].

There are a lot of photosensitizers for PDT, classified into porphyrins, chlorins, dyes (e.g., phenothiazinium salts, rose bengal, squaraines), and recently, nature-inspired PSs.

In radiotherapy, the cellular DNA is the target, but almost all PSs will deteriorate other distinct subcellular entities, which makes the distinction between various PSs applied in PDT.

The perfect PS should accumulate, especially in unhealthy cells, and when light is applied, it should produce toxic species that will destroy the target tissue. Characteristics of an ideal PS are the following: high absorption with maximum absorption coefficient in longer wavelengths, close to the red/infrared spectrum with deeper penetration, to make it possible to deal with grand tumors; better adhesion and fixation in unhealthy tissues than in normal ones; reliable and easy dissoluble in biological systems, permitting intravenous management and fast elimination from the organism after PDT, without residual sensitivity of the skin to light or long-term photosensitization; very good chemical balance and insignificant toxic effect on cells in the absence of light or in low darkness; long triplet lifespan and high triplet state effect, as well as high efficiency for triplet formation and singlet oxygen generation; reduced photobleaching and genuine fluorescence, low production price [6,23,24,25,26].

**Figure 2:**
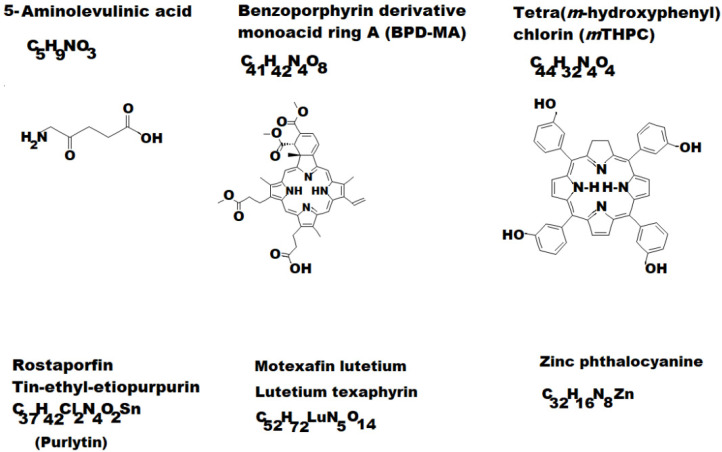
Some examples of second-generation photosensitizers (PSs).

During development, there were several generations of PS, such as:

- the first generation (HpD and Photofrin) had poor absorption in the red visible range, limited applications and an unpleasant side effect, the residual sensitivity of the skin;
- the second generation (see some examples depicted in Figure 2) allowed a much more accelerated development of PDT (5-Aminolaevulinic acid (ALA); Benzoporphyrin derivative monoacid ring A (BPD-MA) or Verteporfin; Chlorins sold as Purlytin; Tetra(*m*-hydroxyphenyl)chlorin (*m*THPC) or Foscan; Lutetium texaphyrin with tradename Lutex or Lutrin; 9-Acetoxy-2,7,12,17-tetrakis-(β-methoxyethyl)-porphycene or ATMPn; Zinc phthalocyanine (CGP55847); Naphthalocyanines (NCs) and Porphyrin-type Chromophores (PC) with modified marginal operation by different functional groups, such as nitrophenyl, aminophenyl, hydroxyphenyl, pyridiniumyl derivatives etc. [7,27,28,29].

Third generation PSs include antibody-directed photosensitizers, such as, for example, the monoclonal antibodies for rising specificity and enhanced capacity of action, better pharmacokinetics, improved function, selective targeted and delivery etc., and support the hope for a future better featured PDT [28,30].

The use of natural compounds has been approached as a new trend in photodynamic therapy, and the natural photosensitizers investigated coupled with the latest nanotechnologies have been proven to be effective in the clinical practice, as is the case with curcumin.

**Figure 3:**
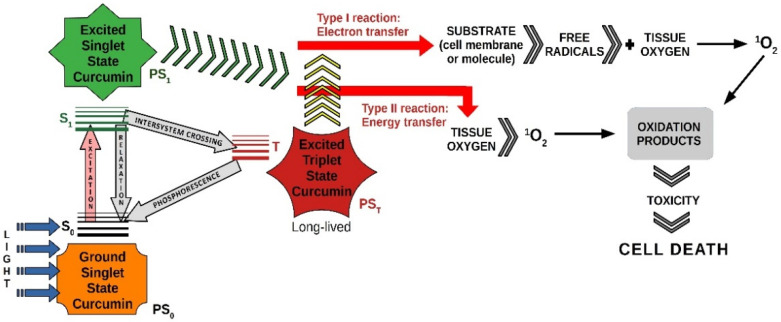
PDT with blue light and curcumin.

An illustrative diagram of photodynamic therapy using curcumin as a photosensitizer is shown in Figure 3.

The use of nanotechnologies in recent years has brought significant improvements in the pharmaceutical industry through the discovery of nanoparticles, extraordinary innovations have been made for the production and delivery of the active principles of new drugs. Recent studies have shown another way to use curcumin to increase its bioavailability, plasma concentration and ability to penetrate and concentrate inside cells.

There are already many techniques for nano formulations, biomaterials and types of nanoparticles suitable for loading curcumin, to increase its therapeutic efficacy and to avoid its possible side effects, as follows:

### 2.1. Curcumin-Loaded Liposomes (Lipo-Cur)

Liposomes are spherical vesicles composed of simple or multiple layers that surround aqueous units and have become ideal delivery systems for biologically active substances, because they offer high biocompatibility and biodegradability, high solubility, stability and flexibility, low toxicity, preparation-controlled distribution with specific cell targeting. PEGylated nanoliposomes (attached strands of polyethylene glycol (PEG) to nanoliposomes) were the first nanoparticles approved for nano-drugs by the FDA.

Curcumin is a bioactive agent isolated from *Curcuma longa* rhizomes that, in the last three decades, has reached the peak of research for its biological functions; anti-inflammatory, antioxidant, antimicrobial, antiviral, antimutagenic, antitumor and anti-angiogenic. Liposomes solubilize curcumin and allow its distribution on the aqueous medium and increase its effect for the treatment of various cancers and other diseases.

It has been shown experimentally that blue light stops the multiplication of cancer cells, induces cell death by activating caspase and increasing intracellular reactive oxygen species. A recent study investigated the in vitro effects of curcumin-loaded liposomes at concentrations of 0–100 µmol/L in combination with PDT at 457 nm (blue) and the fluence of 220.2 W/m^2^ on three papilloma virus-associated cell lines, and proved that after 24 h, the blue light activation of curcumin-loaded liposomes led to “a significant reduction in colony formation and migratory abilities, as well as to an increase in tumor cell death” [31].

Curcumin-loaded liposomes (Lipo-cur) and blue light distributed by PDT can achieve excellent bioactivity and strong anticancer activity [31,32,33].

### 2.2. Cur-Loaded Polymeric Micelles

Polymeric micelles have been used as nanodrugs for over 40 years; they are structured on a hydrophilic shell, and hydrophobic core that can be loaded with hydrophobic drugs [34].

Chang et al. studied how Cur-loaded polymeric micelles influenced endocytosis and exocytosis in colon carcinoma cells and proved that curcumin-loaded micelles had an increased stability compared to the unloaded micelles and were rapidly incorporated by the cells within minutes, becoming cytotoxic after 72 h of exposure.

In this experiment, the micelles applied to encapsulate curcumin had a diameter of about 16–46 nm, and when loading curcumin, their size was approximately doubled, with a loading efficiency of 58%. Results showed the importance of micelle size and loading on drug delivery and cytotoxicity [35].

A wide variety of amphiphilic polymers (diblock, triblock, grafted copolymers, etc.), hydrophobic materials and vitamin E, have been produced for the preparation of mycelium, but the most used are polyethylene glycol (PEG), polyvinylpyrrolidone (PVP) and chitosan [36,37].

Liu et al. have developed curcumin-loaded polymeric micelles to surmount the reduced ability of curcumin to be dissolved in water and to achieve better intravenous administration. Cur-loaded polymeric micelles had a very strong antitumor effect, inhibiting tumor growth and spontaneous lung metastases in a model of breast tumor in mice, proving to be a very good option for breast cancer [37].

In order to easily penetrate the cells and increase the therapeutic intake, the micellar surface was modified by adding ligands (epidermal growth factor receptor, folate, etc.) that more quickly recognize the receptors expressed on the surface by cancer cells [38].

### 2.3. Cur-Loaded Polymeric NPs

Nanoparticles are designed at the atomic or molecular level, have a diameter 1000 times smaller than a medium-sized cell in our body and are very valuable for the administration of drugs, because their physical, chemical and biological properties are unique. Curcumin can be encapsulated in a wide variety of nanoparticles based on polymers, solid, magnetic, gold and albumin-based lipids, which increase its solubility, pharmacokinetics, controlled release capacity and exact cell strike [39,40,41,42].

### 2.4. Cur-Loaded Mesoporous Silica NPs

Nanotechnology has overcome conventional concepts and ideas in the pharmaceutical industry. More than 30 years ago, Mobil Corporation first produced mesoporous silica nanoparticles (MSNs). The mesoporous structure is unique, provides chemical stability and high drug loading capacity, biocompatibility, large pore volume, large surface area, controlled release at the target, and low toxicity. In cancer, there are large disorders of the normal structures of the lymphatic and vascular system, so that MSN nanoparticles can more easily penetrate cancer cells through the process of phagocytosis and pinocytosis. The nanoencapsulation of curcumin with silica and chitosan increases the stability of curcumin and increases its cytotoxic activity on cell carcinoma cells [43,44].

### 2.5. Cur-Loaded Protein-Based NPs

Protein-based nanoparticles are widely used as natural biomaterials in the biomedical field because they meet the special qualities of biocompatibility, biodegradability and non-immunogenicity. Albumin from chicken serum or eggs was the most studied protein for obtaining drug-releasing nanostructures. Recently, many types of Cur-loaded protein nanoparticles have emerged, such as bovine serum albumin (BSA), human serum albumin (HSA), ovalbumin (OVA), zein, casein and curcumin–silk fibroin nanoparticles (CM–SF NPs) [32,45,46].

### 2.6. Cur-Loaded Solid Lipid NPs

Solid lipid nanoparticles (SLNs) are colloidal systems made of biodegradable solid lipids used in the pharmaceutical industry, because they perform through physical stability, high biocompatibility and the controlled release of embedded drugs. Cur-loaded solid lipid NPs were investigated in vitro and in vivo, demonstrating good stability and bioavailability, increased absorption in cells, and high anti-cancer efficacy [47,48,49].

### 2.7. Cur-Loaded CDs NPs

Cyclodextrins (CDs) comprise a family of cyclic glucose oligomers, which have excellent biocompatibility properties, complexation with lipophilic structures, very low toxicity, non-immunogenicity and targeted drug release. Cur-loaded CDs NPs have been shown to be highly effective in antioxidant activity, to reduce the oxidative stress associated with various cancer and a preventive role for nosocomial infections [50,51,52].

### 2.8. Cur-Loaded Nanogels

The proposed nanogel formulation of curcumin has several advantages over other delivery systems, because it ensures a higher concentration of the drug at the target sites and can reduce the exposure of curcumin to serum proteins and biological degradation after systemic administration. Multifunctional hybrid nanogels, through their ability to emit fluorescence, can be used for imaging, cell monitoring and, because they have high absorption in the near infrared range, are useful in photothermal conversion, and loaded with curcumin, are aimed at suppressing drug-resistant tumors [53,54,55].

### 2.9. Cur-Loaded Nanocrystals

Nanocrystals, although small particles have a large surface area for loading the hydrophobic drug, and due to their high solubility and saturation capacity, they increase bioavailability and biodistribution.

For example, in a recent study, Wang et al. have prepared curcumin nanocrystals (CNs) with a mean diameter of 15 nm by the quick emulsion freeze-drying method. CNs proved to be effective in drug delivery to selectively deliver Curcumin to cancer cells. CNs were prepared via the oil-in-water emulsion freeze-dried method: firstly, they prepared the emulsion, the oil phase (O) was dichloromethane solution containing 40 mg/mL curcumin, and the water phase (W) was containing 1 wt% Pluronic^®^F-127-COOH aqueous solution, at a volume ratio O/W fixed at 1:20, and the blend was mixed through ultrasonic (100 w, 10 min) emulsification to obtain the emulsion (O/W). Secondly, the above emulsion was further added into 20-fold the volume of the water phase, and the mixture was frozen by liquid nitrogen, followed by freeze-drying using a vacuum freeze-dryer. Finally, CNs suspension was obtained when lyophilized products were dispersed into the water, and free surfactants were removed by ultracentrifugation [56].

Curcumin nanocrystals (CNs), by the ability to avoid absorption in the reticuloendothelial system, prolong the circulation time of curcumin, increase the capacity of permeation and retention, accumulating in large quantities in the tumor [56,57].

### 2.10. Cur-Metal Oxide NPs

Inorganic nanomaterials used as metal nanoparticles (carbon, nanotubes, graphene, minerals and metal oxides) that carry drugs would be more advantageous than organic ones, because it possesses bioavailability, tolerance towards most organic solvents and better stability, increased surface area and porosity, better loading and dispensing capacity of drugs, with lower toxic effects [58].

Nanoconjugated zinc oxide nanoparticles (ZnONP_CS_) with curcumin (ZnONP_CS_-Cur) showed higher cytotoxicity in several cancer cell lines (breast, cervix, osteosarcoma and myeloma) and had anticancer and anti-inflammatory activity in vitro, and therefore, they could be used as possible therapeutic nanoconjugates for future cancer treatments [59].

## 3. Curcumin and Latest Cancer Applications

On 14 December 2020, International Agency for Research Cancer (IARC) released Globocan 2020, which published the latest data on the incidence of cancer, which rose to 19.3 million new cases and 10 million cancer deaths in 2020. According to Globocan 2020, which is a statistical database for IARC on incidence and mortality in 185 countries for 36 types of cancers, it has been estimated that cancer had 19.3 million new cases per year, of which breast cancer was in first place, with about 11.7% new cases, followed by lung cancer 11.4%, colorectal 10%, prostate 7.3%, and followed by stomach cancer at 5.6% [60,61].

The field of cancer research is a dynamic and evolving domain, with a multitude of models and scenarios proposed for examination over the decades on the hidden cause behind tumors development (genetic mutations, microorganisms, metabolic changes and so on), with fluctuating evidence or achievements, whose major goal remains the discovery of new methods and drugs for stopping disease progression and even eradicative therapeutics.

On this line, the cancer stem cell (CSC) model encompasses unusual immortal cells, such as those existing in tumors or blood cancers, similar to regular stem cells, but capable of generating the full range of cells from a given cancer specimen, which, through self-renewal and differentiation into multiple types of cancer cells, are tumorigenic, i.e., they generate recurrences and metastases, and so, supplementary malignancies [62,63].

In cancers that pursue the CSC model, some intracellular pathways may be attacked with natural compounds, such as curcumin or drugs, to overcome the danger of the development of new tumors at a distance. Promoting appropriate CCS-oriented treatments could improve the survival and quality of the life of patients with metastases [25,63,64,65,66].

Very recently, research based on this model shows the effectiveness of curcumin in various forms of cancer [67].

Even though it has reduced bioavailability, being insoluble in water, curcumin has been intensively studied as an authentic polyphenol and practically the main constituent of *Curcuma longa*, for its multiple beneficial effects in the treatment of various inflammatory, auto-immune, degenerative diseases, etc., and going to important applications in cancer, not only for the protective effect, but especially by destroying malignant cells. To overcome this drawback, various water-soluble mixtures have been imagined, such as liposomes or the incorporation of curcumin into micelles at the nanometer scale, with raised assimilations appropriate for cancer studies. The first formulations of curcumin in organic solvents proved to be toxic to living cells, and even with genotoxic capabilities. No investigation with curcumin embedded in the micelles has been planned until not long ago. In a recent experiment, Beltzig et al. comparatively investigated the cytotoxic and genotoxic action of genuine curcumin dissolved in ethanol (Cur-E), or integrated into micelles (Cur-M), and evaluated cell killing, apoptosis, necrosis, senolysis and genotoxicity, on a multitude of elementary and settled cell lines, proving that both formulations reduced viability for all cells in the same dose interval. Cur-E and Cur-M induced apoptosis as a function of dose, without senolytic action. Genotoxic repercussions disappeared in the absence of curcumin, denoting a prompt and full repair of DNA. In every experiment, Cur-E and Cur-M were, to the same extent, dynamic, and had important cytotoxic and genotoxic action, starting with 10 μM. Micelles without curcumin content were fully inoperative. The results proved similar in terms of cytotoxicity and genotoxicity for micellar curcumin as the native one, so the administration of micellar curcumin as a dietary supplement is safe and paves the way for new applications [68].

Major goals of pharmaceutic investigations are the innovative transport/delivery systems of drugs in cancer treatments. Zarrabi et al. have researched the manufacture of a new intelligent biocompatible stealth-nanoliposome to supply curcumin in cancer therapies. Four distinct classes of liposomes (plus or minus pH-sensitive polymeric film) were obtained by the Mozafari process, and then investigated by multiple trials. The embarkation and deliverance of curcumin were assessed at two different pH values, 7.4 and 6.6, but also the cytotoxicity of the specimens. The optimal average size for the smart stealth-liposome was 40 nm, and the efficacy of the drug’s catch was about 84%, comparatively with 50 nm and only 74% performance by uncovered liposomes. Nano-carrier discharge from the stealth-liposome was better directed than in the uncovered. Experiments have shown the toxicity of drug’s nanocarriers on malignancies. We could conclude that soon, the pH-sensitive intelligent stealth nanoliposome may become a true aspirant in cancer treatments [69].

Resistance to medicine and bad outcome in some cancer cases is often due to the hyperactivation of NRF2, a group of transcription factors, i.e., the nuclear factor erythroid 2 p45, detected in some tumors. It was demonstrated that curcumin can induce either cytoprotection or tumor growth by activating NRF2, as a function of the phase of the malignancy. Garufi et al. highlighted the anticancer effects through manifold molecular processes related to curcumin, and recently explored the fundamental molecular sequence of steps linked to making operative NRF2 by the zinc-curcumin [Zn (II) -curc] complex. Indeed, the therapy with Zn (II)–curc raised the NRF2 proteins concentrations and their connections, the heme oxygenase-1 (HO-1) and p62/SQSTM1, while particularly decreased the levels of Keap1 (Kelch-like ECH-associated protein 1), which stopped the NRF2 in all the investigated malignant cell lines. The inhibition of NRF2 or p62/SQSTM1 with distinctive siRNA proved the existence of the communication channel between the two molecules, and that the easily disassemble of any molecule surged the killing of cancer cells by Zn (II)–curc, a fact that could be implemented in the future to improve the receptivity to tumors treatment by this method [70].

Curcumin displays multiple proven effects on cells; for example, an inhibitive action on thrombocytes, but not known if it is owed to thrombocyte apoptosis or to pro-coagulant platelet organization.

Recently, Rukoyatkina et al. reported that curcumin did not initiate caspase 3—relying on the apoptosis of human thrombocytes, but led to the organization of pro-coagulant thrombocytes. At 5 µM concentration, the effect increased, but at ten times’ higher concentration, the thrombocytes apoptosis was stopped by the suppression of ABT-737 (small molecule drug that inhibits Bcl-2 and Bcl-xL) that was interfered with thrombin production.

Curcumin did not alter thrombocytes’ ability to survive at low concentrations but decreased it by 17% at higher concentrations. Autophagy caused by curcumin in human thrombocytes was accompanied by the operative configuration of adenosine monophosphate kinase (AMP), and the cessation of protein kinase B function. Curcumin could block the P-glycoprotein (P-gp) in tumors, and therefore defeat the manifold medicines resistance, and likewise could also stop the thrombocytes P-gp action. The effects of curcumin on human thrombocytes are due to complex processes through pro-coagulant thrombocytes organization, and so it can support pro—or against caspase—subordinate thrombocyte’s death, but only in distinctive cases [71].

Systematically checking the abnormal functioning of cells, tissues or organs in the inceptive steps of the initiation of malignant processes and the monitorization of the cellular oxygenation is of maximum significance, both for the fundamental applications, but also in the practical medical ones.

A non-invasive modality for both the in vivo and in vitro assessment of cellular oxygenation is evaluating the lifespan of the luminescence of molecular sensors, but still very difficult in the case of increased oxidative stress.

Molecular probes, such as mitochondrial probes or [Ru (Phen)3]^2+^ state-of-the-art, intact cell phosphorescence imaging technologies applied by Huntosova et al., in a model of chorioallantoic membrane (CAM), offer reduced phototoxicities and could also be applied in curcumin cancer therapy in tumors originating from the neuroglia of the brain or spinal cord. These results could be useful and universalized for the evaluation of tissue oxygenation as an advanced and original method, based on the analogies between diverse interacting biological factors, especially in cancer therapies that deal with metabolic or oxygen changes, glucose and lipid loss, and so on [72].

Important attempts to improve the potency of targeted drug carriers in the lung cancer were made, but the prognosis is still very poor, with only 15% survivors, 5 years after identification. The best choice for the direct administration of chemotherapy to the lungs would be the inhalation formulation.

Currently, this type of formulation to accomplish successfully, at the same time, a significant dose of specific chemicals that are selectively destructive to malignant cells and tissues in the solid tumor and to function with reduced local lung toxicity, is still an aim, as only 10–30% of lung chemotherapy nowadays already has the quality of being toxic [73].

Lee et al. imagined a dry powder easy to inhale (DPI) holding a chemotherapeutic agent (paclitaxel, PTX) and the native antioxidant curcumin (CUR) that defends the healthy cells to be damaged during direct lung transfer chemotherapy. Grinding CUR and PTX in co-jet as aerosol formulation, with more than 60% of very fine particles and a fit mass median aerodynamic diameter, exhibits an important cytotoxic effect for lung tumors, giving rise to apoptosis/necrotic cell killing, extending mitochondrial oxidative stress (ROS), depolarizing the mitochondria membranes, and decreasing the ATP in malignant cells. Incorporating CUR is decisive for correcting the cytotoxic effects of PTX against healthy cells and depends on dose, providing an easy and efficient DPI formulation with special discriminating cytotoxicity in lung malignancies [73].

In another experiment concerning lung cancer, Wan Mohd Tajuddin et al. studied the diarylpentanoid (DAP), changed the structure analog from the genuine curcumin, and proved to improve anticancer effects in different forms of malignancies, by comparing the outcomes (toxic impact, proliferative and apoptotic action) on two subtypes of non-small cell lung cancer (NSCLC) cells: the squamous cell carcinoma (NCI-H520) and the adenocarcinoma (NCI-H23). The gene expression to reveal the main signaling pathways, the targeted genes, the cytotoxicity screening, the anti-proliferative action, as well as the apoptosis linked to the rise in caspase-3 activity and decrease in Bcl-2 protein concentration were investigated and proved to be function of dose and time in all studied cells. This new compound, derived from curcumin, should be henceforth investigated as a possible representative anticancer drug for NSCLC cancer treatment [74].

## 4. Effects of Curcumin and PDT in Various Forms of Cancer

### 4.1. Breast Cancer

Breast cancer at onset has no clinical symptoms, because it initially affects the glandular epithelium of the ducts or lobe structure, but over the years, it can progress and invade the surrounding breast tissue and lymph nodes or various organs; therefore, if a woman dies, the cause is multiple distant metastases. Breast cancer can occur in women right after puberty, or at different stages of life, and the number of cases has increased year on year, becoming the most common form of cancer worldwide. According to World Health Organization (WHO) publications, as of 2020, 2.3 million women have been confirmed with breast cancer, of which 685,000 deaths have occurred worldwide, and an impressive number have been left with various disabilities in daily life [75].

It is currently known that breast cancer can be classified into three subtypes:

- type 1 breast cancer with estrogen receptor (ER+) positive hormone or progesterone receptor (PR+) positive; this type responds to hormonal treatment.
- type 2 breast cancer with a positive test for the human epidermal growth factor receptor 2 (HER2), a protein which fosters the development of cancer cells, and may respond to HER2-targeted treatments.
- type 3 breast cancer is the one known as triple negative breast cancer (TNBC), because here we do not find ER, PR or HER2. This type of cancer is the most difficult to treat with pharmacological means that have already become classic. Various drugs [Sacituzumab govitecan (Trodelvy)] and immunotherapeutic products, [Pembrolizumab (Keytruda), PARP inhibitors], are being tested in combination with conventional chemotherapy for this type of breast cancer [76].

Although the breast cancer therapy available today (anticancer chemotherapeutic drugs, antihormones, biologics and radiation therapy) has very good results on early detected primary tumor, many deaths still occur due to recurrences and multisystemic metastases. In this pathology, it has been shown that a type of cancer cells, like normal stem cells, has a strong potential for multiplication, self-renewal, differentiation, and their oncogenicity can underlie recurrences and metastases [77].

Radiation therapy is one of the standard treatment methods for breast cancer; applied in the early stages, it can decrease mastectomy surgery; in later stages, it can reduce the risks of recurrence, and in advanced stages, it can prolong the patient’s life [75].

However, this method still has many disadvantages, in that the tumor may become radioresistant, and cancer and metastases may recur.

Targeting breast cancer stem cells is the clue for ameliorating the results of breast cancer radiotherapy.

Yang et al. investigated the effects of curcumin combined with glucose nano-gold particles (Glu-GNPs), for a decrease in radiotherapy resistance activity, by targeting MCF-7 and MDA-MB-231 breast cancer stem-like cells (BCSCs), in order to increase apoptotic, colony-forming and antiproliferative activity. Irradiation was carried out with a 6 MV X-ray at a total dose of 0 and 4 Gy; the dose rate was 2 Gy/min, for two minutes, the depth of penetration was 2 cm, and the irradiation distance of 50 cm. The authors demonstrated that curcumin, combined with Glu-GNPs, increased the ROS level of mammals MCF-7 and MDA-MB-231 in hypoxic conditions by inhibiting the factor-1alpha (HIF-1alpha) (an oxygen-sensitive transcriptional activator) and the heat shock protein 90 (HSP90) activities, ameliorating the apoptotic activity of tumor stem cells, and thus, it has increased the sensitivity to radiation therapy [77].

Minafra et al. performed a study to evaluate in vitro the bioavailability and the radiosensitizing effects of curcumin-loaded solid nanoparticles (Cur-SLN) on three breast cell lines, non-tumorigenic MCF10A and tumorigenic cell lines MCF7 and MDA-MB-231 BC, exposed to irradiation. A multi- “omic” assay was used to clarify the radiosensitizing action of Cur-SLN by microarray and metabolomic exploration techniques. The authors demonstrated the antioxidant, radiosensitizing and anti-tumor capacity of Cur-SLN through a transcriptomic and metabolomic analysis [78].

The conventional treatment protocol for breast cancer includes breast removal surgery, X-ray therapy, and the administration of specific drugs, i.e., chemotherapy. Drugs administered for these cancers have many side effects, negatively affecting patients’ quality of life, which has required the discovery of new effective, but less toxic treatments. In recent decades there has been a growing trend to find natural remedies with anti-tumor potential, including curcumin and its derivatives.

Research to date has shown that curcumin has anti-neoplastic properties through various mechanisms, including the following: inhibition of endothelial growth factor [79], lipoxygenase enzyme (LOX) pathway [80], blocking the activity of NF-kB and Wnt signaling pathways [81,82,83], stops cell cycle and p53-dependent apoptosis, disrupts the expression of signaling protein kinase B (Akt) and phosphatidylinositol 3-kinase (PI3K) [84,85].

Experimental studies have shown that curcumin can inhibit EZH2 (enhancer of zest homolog-2), which is a histone methyltransferase that catalyzes the trimethylation of histone H3 in lys 27 (H3K27me3), is found in an increased amount in human cancer, has an oncogenic, metastatic role, and influences drug resistance. It was postulated that, on the EZH2, miR-375, FOXO1 and p53 axis, special processes with direct impact in the proliferation of the breast tumor could take place [86,87].

Gallardo et al. showed, in a study on MCF-10F and MDA-MB-231 cell lines of human breast cancer, that curcumin can modulate the expression of miR-34a and Rho-A, which reduces cancer progression, metastasis, and increases the sensitivity of anti-cancer drugs [88].

The chemical structure of curcumin is responsible for many of its biological and pharmacological activities, as low solubility in aqueous media, poor bioactive absorption, physicochemical instability, rapid metabolism, sensitivity to alkaline environment, which restricts its clinical scope [89,90].

With the patenting of curcumin nanoencapsulations, these impediments have been overcome. There are currently several nanoencapsulation techniques, but ionic gelation and antisolvent precipitation are in vogue. The products used for nanoformulations currently include: liposomes, polymers, nanoparticles, conjugates, solid dispersions, cyclodextrins, micelles, nanospheres and microcapsules and other various nanoformulations [42,90,91].

Due to the maximum wavelength of blue light absorption (408–434 nm), curcumin is used as a natural photosensitizer for PDT applications in various medical, antimicrobial, antiviral and antitumor fields [92,93].

The mechanisms by which PDT can eradicate tumor tissue are synthesized in the following modes of action: initially, it will locate and activate PS in the tumor area, which will release ROS with the potential to destroy neoplastic cells; the second mechanism would be that PDT disrupts the usual supply of oxygen and nutrients by compromising vascularity; the last postulated mechanism is the activation of the immune system, which triggers an inflammatory process against tumor structures.

Sun et al. studied experimentally, on 4T1 mouse breast cells, the effects of carrier-free curcumin nanodrugs (Cur NDs) used in conjunction with PDT administered by a device that emitted blue light at a power of 640 mW on a wavelength of 450 nm. Cur NDs were prepared without using any toxic solvents through an easy and green reprecipitation method, as follows: 1 mg/mL Cur was dissolved in ethyl alcohol; afterwards, 1 mL of the solution was rapidly injected into 20 mL of high-purity water under robust stirring. NDs were purified and concentrated by ultrafiltration, and at the end, the Cur NDs were achieved by lyophilization. In vitro irradiation (λ = 450 nm, 640 mW, 1 min) of 4T1 cells after 24 h of incubation significantly increased the expression of p-JNK, Bax and cleaved caspase-3, with the generation of a large amount of ROS, activation of MAPKs with induction of apoptosis, and reduced the cells viability. The study demonstrates that Cur NDs is a valuable photosensitizer for PDT in eradicating breast cancer, and with very good prospects for use in the clinical practice [94].

Because curcumin has a low solubility, nanoemulsions and microemulsions as administration systems with dimensions between 100–300 nm facilitate an expansion of bioavailability, the therapeutic window, and even a controlled delivery to the desired area [95,96,97].

Machado et al. investigated the effect of curcumin-nanoemulsion (CNE), a new and well-designed drug delivery system (DDS+) molecule, as a novel photosensitizer in the photodynamic therapy of breast cancer on MCF-7 cell model. CNE was achieved by interfacial pre-polymer deposition and spontaneous nanoemulsification. Curcumin was set up in an oil phase at 0.1 mg/mL. Organic phase (acetone) was obtained from medium-chain-triglycerides, natural soy phospholipids. This was added into the aqueous phase containing an anionic surfactant, poloxamer 188. Organic solvent was taken off through rota-evaporation. The authors revealed that curcumin encapsulation in lipid nanoparticles increased the concentration of this compound, its biological effects, solubility and bioavailability, without substantially affecting the cell viability of HFF-1 and MCF-7 cells. After incubating HFF-1 cells and MCF-7 cells and applying two doses of 80 J/cm^2^ with a laser device at 440 nm, the mortality rate of breast carcinoma cells was 65% and 90%, respectively, demonstrating the value of CNE as photosensitizer [98].

Curcumin, a component derived from the dried rhizomes of *Curcuma Longa*, is the most requested phytochemical component for cancer therapy, and is therefore considered the “magic molecule” [99,100].

Remote spread of primary malignancy is the cause of multiple cancer deaths. To date, the entire arsenal of available drugs and treatments are still insufficient to manage the metastases that are forming. Therefore, maximum attention is directed to the prevention of metastases by targeting circulating metastatic tumor cells.

A lot of circulating tumor cells (CTCs) will perish in the blood circulating through the body, but a small number will most likely generate distant metastases, and thus a negative prognosis for the patient. These stem-type CTCs can escape immune surveillance and show a high ability to withstand treatment. The latest strategies should address metastatic CTCs and their progenitors based on their molecular and cellular characteristics, to expand current plans for the successful prevention of metastases [101].

In a recent study in this significant direction, Raschpichler et al. advanced an in vitro exploratory model to mimic the circulation of CTCs, and to render them inactive by PDT and curcumin-loaded poly (lactic-co-glycolic acid) nanoparticles. Researchers obtained after blue laser irradiation (30 min, λ = 447 nm, output power 100 mW), an important decrease in the viability of MDA-MB-231 breast cancer cells under flow conditions. Accumulation of curcumin on cell membranes and high fluorescence signal after irradiation was detected by laser scanning confocal microscopy. PDT determined alterations in the cancer cells, i.e., apoptosis and prompt necrosis, as a function of time, and during laser activation of curcumin nanoparticles, a change in blue absorption spectra and a decrease in total curcumin occurred. Findings are incentive for the extension of these in vitro CTCs studies to in vivo experiments, so that PDT becomes an advanced action plan for future clinical applications for metastasis prevention [102].

The anti-cancer activity of curcumin by formulation as nanostructured lipid carriers (NLCs) using solid lipids (glyceryl monooleate, glyceryl dibehenate, glyceryl distearate) and olive oil as a natural liquid lipid, was tested in vitro on a breast cancer cell line. The curcumin thus prepared has been used as a photosensitizer for PDT in MCF-7 breast cancer cells. The loading of CUR into NLCs increased the penetrating power into cancer cells and the cytotoxic potential in both dark and light conditions [103].

Approaching cancer therapy with current means brings a major drawback, namely the deterioration of healthy tissue in the vicinity of the tumor. To avoid these major problems, the association between PDT and photothermal therapy (PTT) has aroused the interest of researchers in the therapy of a wide range of diseases, including cancer. Very good results with reduced invasive effect and negligible side effects were obtained by combining PTT that releases heat, and PDT that not only produces ROS, but also promotes the release of products with multiple antitumor immunity capacity, in order to dissipate tumors. Phototherapy using nanoparticles can target and destroy undetectable metastatic tumors, restore the anti-tumor capacity of drugs and modulate the immune system in the tumor microenvironment [104,105,106,107].

The association between PTT and PDT has the advantage of an excellent curative result by eliminating tumor cells, as well as by immunomodulating the apoptotic and inflammatory responses of tumor cells.

An additional barrier of the immune control point to the photo treatment could increase the antitumor effects, generating more CD4 + and CD8 + T cells in the tumor [108].

An in vivo experiment on Female Balb/c mice tumorized with 4T1 cells has investigated the antitumor effects of curcumin loaded with Fe_3_O_4_–SiO_2_ nanoparticles, i.e., a dual-functioned nanocomposite (NC), and also PDT and PTT. After tumorization, the mice were divided into six groups: control group injected with phosphate buffer saline (PBS); group II with CUR and irradiation with blue diode laser at 450 nm, intensity of 150 mW/cm^2^ for 3 min (CUR and PDT group); group III, PBS injection plus irradiation with blue diode laser for 3 min followed by NIR laser with intensity of 0.5 W/cm^2^ for 7 min, i.e., the blue + NIR (near-infrared) laser group; group IV, nanocomposite injection (NC group); group V with NC plus irradiation with NIR laser at 808 nm for 7 min (NC and PTT); and the last group with NC + PDT + PTT. The treatment protocol combining NC and PDT together with PTT on the highly invasive triple-negative breast cancer model used in this study could be considered for the replacement of chemotherapy in these types of cancer.

Results of the study revealed that in the group treated with NC and PDT together with PTT, the tumor volume decreased significantly, and the expression of proapoptotic proteins Bax and Caspase 3 increased meaningfully compared to the control group, without weight loss, and no adverse effects on vital organs (liver and lung) in the mice autopsy image [109].

In an in vitro study, Halevas et al. reported that the gallium curcumin complex used as a photosensitizer in photodynamic therapy on human breast MCF-7 adenocarcinoma cells, increased intracellular ROS levels, and significantly decreased the number of cancer cells compared to simple curcumin. New curcumin-Ga complex did not show dark toxicity at low concentrations against MCF-7 breast cancer cells, and the decrease in cell survival was dependent on the laser dose [110].

Table 1 summarizes aspects of recent research that have shown that curcumin nano formulations, together with PDT, can contribute to the treatment of breast cancer by strong penetration into cancer cells, high cytotoxic potential, decreased tumor volume, prevention of metastases, without weight loss, and without adverse effects on vital organs (liver and lungs) in experimental animals, with prospects for future clinical applications [94,98,102,103,109,110].

### 4.2. Gynecologic Cancers

Cancer worldwide is a major health problem, and one of the most common causes of death [111]. According to statistics from the last two decades, the incidence of cancer in women has been steadily increasing by about 0.5% per year for breast cancers, and about 1% for uterine cancer, due to the reduction in the number of fertile women and weight gain [112,113,114].

Over the past three decades, the survival rate of cancer patients has increased, except for the cancers of the uterine cervix and of the uterine corpus [115].

Cervical cancer, with almost 0.6 million cases and 0.3 million deaths per year in 2018, ranked fourth in the world in terms of incidence and mortality caused by cancer after breast cancer (2.1 million cases), colorectal cancer (0.8 million cases) and lung cancer (0.7 million cases) [116].

Today, special efforts are being made to prevent, detect early and implement new therapeutic strategies to improve the efficacy and safety of chemotherapy and radiotherapy, prevent recurrences, metastases and increase patient survival. PDT, together with various curcumin nanoformulations, are promising treatment modalities for a wide range of cancers, such as cervical cancer.

Nanoemulsion-curcumin behaved as a photosensitizing drug in PDT, generating a high phototoxic effect, with less than 5% of viability in the experiment with cervical carcinoma cell lines (CasKi and SiHa) and human keratinocytes spontaneously immortalized cell line (HaCa). In an experiment done by de Matos et al., increased activity of the enzymes caspase 3 and caspase 4 was observed, suggesting that cell death occurred by apoptosis. The authors propose the use of curcumin-nanoemulsion and PDT through an in situ optic fiber, as an alternative treatment in cervical cancer [117].

A very important issue in PDT is to avoid the accumulation of PS in healthy tissues and prevent unwanted effects, which can be solved by using encapsulated PS in polymeric nanoparticles, in which the active product is protected from degradation by the physiological environment; an example is the case of poly (lactic-co-glycolic acid) (PLGA), recognized as one of the most valuable drug-carrying polymers (DCs) that is used to obtain high quality products. PLGA polymer is approved as a biocompatible and biodegradable polymer by the FDA and the European Medicines Agency (EMA) [118,119,120].

Duse et al. published the results of in vitro irradiation research (457 nm LED with a radiation flux of 8.6J/cm^2^) on the human ovarian adenocarcinoma cell line SK-OV-3, using a photosensitizer with biodegradable PLGA nanoparticles loaded with curcumin (CUR-NP). In this study, we analyzed the effect of nanoformulation on human erythrocytes, by haemolysis test and blood clotting, which showed that there was a slight increase in haemolysis and improved serum stability, assuming that there is an interaction of curcumin with intrinsic proteins or coagulation factors. The authors showed that the nanoformulation allowed the use of higher amounts of curcumin, which showed cytotoxic effects on tumor cells after the administration of PDT at low intensity, thus selectively inhibiting tumor growth, and believe that LEDs provide an economic and technical advantage over laser devices [121].

Curcumin, as a special low-toxicity photosensitizer, has higher pro-apoptotic potency when combined with PDT. However, the mechanisms of action are still poorly understood. He et al. investigated the results of the combination of different concentrations of curcumin mediated PDT with/without Notch receptor blocker (DAPT), after 180 s of irradiation with 445nm laser at a dose of 100 J/cm^2^, used together with curcumin, on the survival rate of cervical cancer Me180 cells in female BALB/c mouse. Following the administration of curcumin with PDT plus DAPT, a synergistic interaction occurred that significantly increased the overall rate of cell mortality. The authors claim that this combination could inhibit Notch-1 expression and downstream protein synthesis in in vitro and in vivo cervical cancer. Notch-1, an advanced preserved signaling pathway that controls interactions between adjoining cells and NF-κB, could be the targets of the curcumin-PDT combination in the success of cervical cancer therapy in women. This can be explained by the fact that Notch-1 activation is associated with the onset and development of cervical cancer [122].

Figuratively speaking of Notch, we can consider that it belongs to the group of arbitrators who decide the fate of the cell; in fact, it modulates the balance between differentiation and multiplication of cells. The Notch pathway has a very important role in the evolution of breast, cervix, ovary, and uterine endometrium epithelial tissues, and is frequently involved in the appearance and expansion of cancers [123].

### 4.3. Skin Cancer

Human skin consists of three layers of cells superimposed from depth to the surface, as follows: epidermis, dermis, and hypodermis. The epidermis consists of five types of overlapping cells, one on top of the other, from the depth to the surface in the basal or germinal layer—spiny, granular, lucidum and horny. All the layers come from the germinal one, whose cells, as they multiply, are pushed to the surface, changing in 26–28 days their shape and structure, and then they will be eliminated as dead cells in the form of barely visible scales. Melanocytes are dermal cells that secrete melanin, a pigment that tans the skin under the action of the sun, and the corpuscles Meissner, Ruffini, Krause, Vater-Pacini and Merkel give the skin the ability to perceive and transmit the proprioceptive information to the brain. If these cells undergo pathological changes, they will give rise to various forms of skin cancer. Each type of skin cancer has a potential for severity, so it must be detected early and treated accordingly. The most common types of skin cancer include the following: basal cell carcinoma, recurrent basal cell carcinoma, squamous cell carcinoma, melanoma, Merkel cell carcinoma, and other types of rare skin cancers [124].

According to data published by the American Cancer Society, about 5.4 million basal and squamous cell skin cancers are diagnosed each year in the US, of which approximately 3.3 million American patients have basal cell carcinoma, meaning that 8 out of 10 have the malignant cells in the basal layer.

Basal cell carcinoma develops in the basal layer of the epidermis, has a tropism for the neck and head regions that are more frequently exposed to the sun, but can evolve elsewhere and over time metastasize to the loco-regional ganglia [125].

Basal cell carcinoma can recur in the same area as before, or in another zone of the body; it has a risk of recurrence of up to 50% after 5 years of diagnosed primary cancer. The risk of recurrence is higher in patients with a personal history of eczema, dry skin, prolonged exposure to the sun or tanning devices with UV light, who have had deep skin carcinoma or a size greater than 2 cm.

Squamous cell skin cancer grows slowly, metastasizes less often, but can invade deep into the skin. This type of skin cancer develops from the flat squamous cells that form the superficial layer of the epidermis, and occurs mainly in the region of the neck, face, ears, external genitalia, dorsal area of the hands, etc. [123].

The exact number of the most common types of basal cell and squamous cell cancer (i.e., keratinocyte carcinoma or KC), known as non-melanoma skin cancer, is not known exactly, because it should not be reported in cancer registries [126].

Among Caucasian populations, squamous cell carcinoma (SCC) of the skin, also known as cutaneous squamous cell carcinoma (cSCC), accounts for 20% of all malignant skin tumors [127].

The incidence of squamous cell carcinoma (SCC) increased in recent decades by 10% per year, so today, the ratio is 1:1 compared to basal cell carcinoma (BCC) in populations in Australia and the US. The most important risk factors for disease or recurrence are chronic sun exposure, ultraviolet A (UVA) radiation, patients using immunosuppressive drugs, those with organ transplants, and the HIV-positive. The disease is more common in the elderly and men. The diagnosis of cSCC can be made especially based on the clinical aspect, which must be confirmed by histopathological examination to anticipate the correct prognosis and the management of therapy. The first intention treatment is the one of complete surgical excision, with histopathological control of the excision edges. When lymph nodes are contained by cSCC, a regional dissection of the lymph nodes will be performed, followed by radiotherapy and various chemotherapeutic agents, and more recently, photodynamic therapy and epidermal growth factor receptor (EGFR) inhibitors [128,129,130,131,132].

Response to treatment is usually good; however, a subtype in the cSCC category needs special monitoring because it has a much higher risk of local recurrence, locoregional metastasis, or distant metastasis and death [133,134].

Although primary cSCC is not a fatal tumor, it can raise major cosmetic problems caused by localization in the face area (surgery can be disfiguring), increased morbidity, and high costs, which will place a significant burden on the public health system [124,131].

The prognosis can be very good, with a 90% survival rate for 10 years, when the disease is in its early stages; if the cancer is extensive or metastasized, survival is reduced to 15.3 months on average, making it the second leading cause of death from cSCC after melanoma in skin cancers [135,136].

Surgical treatment in this type of cancer is the first choice; however, if the tumor is enlarged, its resection will lead to an unsightly scar with a strong psychological impact. Chemotherapy has major disadvantages due to haematological, gastrointestinal, hepatic, or renal side effects, the emergence of drug resistance, and necrosis, fibrosis, secondary tumor formation on long-term administration; after radiotherapy, free radicals can be released that cause local inflammations, dermatitis, ulcers, etc. [137,138].

Given these aspects, it is very important to be found and select effective and safe means of treatment in skin cancer. In recent decades, PDT has established its position in the treatment protocol with predilection for skin cancer, including basal cell carcinoma, recurrent basal cell carcinoma, squamous cell carcinoma, Bowen’s disease, actinic keratosis, etc. Depending on the photosensitizer and the light source, PDT triggers oxidative stress by generating ROS in cancer cells and produces their apoptosis [139,140,141,142,143,144].

Xin Y. et al. investigated the apoptotic effects and molecular mechanisms of action of a treatment combination of ultraviolet B radiation and demethoxycurcumin (DMC) in vitro on A431 and HaCaT cells. The association between demethoxycurcumin as a photosensitizer and ultraviolet B radiation induced apoptosis in vitro in A431 and HaCaT cells, by activating p53 and caspase pathways, increasing Bax and p-p65 expression and suppressing Bcl-2, Mcl-1 and NF-κB pathway. At the same time, high levels of reactive oxygen species were observed, along with the significant depolarization of the mitochondrial membrane [145].

Abdel Fadeel et al. demonstrated the beneficial effects of PDT with violet light (410 nm) and Cur-loaded PEGylated lipid nanoparticles in an in vitro study with increased cytotoxicity in cell culture, human squamous cell carcinoma cell line (A431), and in vivo skin carcinoma in mice [146].

The hypothesis of this study is supported by other researchers [147,148].

Cur can produce higher cytotoxicity when charged on a nanocarrier and, in addition, this quality can be increased by exposure to blue light radiation, therefore, together they will generate ROS that affect the activity of components cell and mitochondrial membrane that will lead to the apoptosis of cancer cells.

In patients with advanced cancer who are malnourished and stay in bed longer, but also in other categories of people who do not mobilize, especially in the elderly, the skin may degrade due to local compression, ischemic and reperfusion disorders [149].

Skin in that area initially becomes erythematous, then necrosis follows and bed-sores or pressure ulcers form. A recently published meta-analysis showed that, in adults, pressure ulcer (PU) has a prevalence of 12.8%, and the incidence rate is 5.4% per 10,000 patients per day, and the rate of PU acquired in hospital was 8.4% [150].

In addition to the high costs for patients and health services, these injuries can even be fatal through local and systemic infections. The conventional treatments with antibiotics, surgical measures, ultrasound, electromagnetic or genetic therapies have failed to completely cure PU in an advanced stage of evolution.

To avoid all these aspects, it seems that a much more beneficial option would be that of the concomitant use of laser radiation, together with curcumin.

The results of studies published so far on the concomitant use of curcumin and laser radiation demonstrate two interesting aspects, namely: laser light [151,152], can excessively increase ROS production, while curcumin is able to inhibit oxidative stress by reducing the activation of ROS-generating enzymes or by eliminating free radicals; these phenomena are very useful for the purpose of the therapy, when it is desired to induce apoptosis in skin cancer or to accelerate the healing process of PU [153,154].

Ebrahiminaseri et al., in an in vitro experiment on mouse MEFs cells using the association between dendrosomal nanocurcumin (DNC) and laser therapy at a dose of 0.95 J/cm^2^, demonstrated the significant migration of MEF cells in the denuded area, increase in the S-phase cell population and the growth factors (TGF-β, VEGF) and decrease in the cell population in the GF/G1 phase and of the pro-inflammatory cytokines (TNF-α, IL-6). This combined therapy (DNC + PDT) also highlighted the modulating role of nanocurcumin on the production and excessive accumulation of ROS generated by the action of the laser. In conclusion, the authors consider that additional in vivo studies are needed to confirm the hypothesis that the combined treatment of DNC simultaneously with PDT (450 nm) may favor the wound healing process [155].

Malignant melanoma is an aggressive form of cancer that results from the degeneration of melanocytes, cells of neuroectodermal origin with the role of melanin secretion as a protective response of the skin against the harmful action of ultraviolet sunlight.

Incidence of melanoma [156], mainly metastatic melanoma, is constantly increasing, especially after global warming.

Although current treatment is well established by surgical excision followed by chemotherapy, immunotherapy, recently based on inhibitors of immune checkpoints, small molecule-targeted therapy and oncolytic viral therapy, which have led to an increase in the survival rate of patients at 5 years; however, the incidence of metastases, the high number of deaths and the side effects of drugs require studies to find new therapeutic ways [157,158].

Szlasa et al., in an in vitro study focusing on the anticancer effect of PDT together with curcumin, revealed an increase in the number of apoptotic and necrotic cells by the overexpression of caspase-3, DNA cleavage and reorganization of the actin cytoskeleton, compared to incubation without irradiation. Fibroblasts have been less influenced by this treatment, which seems beneficial for the increased ability to regenerate irradiated skin areas. PDT, together with curcumin, can be an effective way to induce apoptosis in melanoma [159].

Nanoparticles of selenium-polyethylene glycol-curcumin (Se-PEG-Cur) used in an experimental study on melanoma cancer cells as a photosensitizer for phototherapy and sonotherapy greatly increased intracellular ROS levels and cytotoxicity and decreased cell viability [160].

The liposomal formulation of curcumin has been shown to be more effective in killing malignant cells in several studies. The increase in ROS generation and the photo-destructive effects of PDT, together with turmeric tetraether liposomes on microvasculature, were observed by Duse et al. in an in vivo study using the chick chorioallantoic membrane model [121].

Research group led by Vetha et al. reported apoptosis of A549 cancer cells in vitro, by generating singlet oxygen, increasing intracellular ROS levels and activating Caspase-3, by using small CUR molecules, encapsulated in liposome nanoparticles (LIP-CUR) coupled with PDT via emitting diodes blue light (BLED) [161].

In an experimental study, Woźniak et al. demonstrated a significant improvement in the bioavailability and stability of encapsulated liposomal curcumin as a potent apoptotic photosensitizer in squamous cell carcinoma (SCC-25) and melanoma (MugMel2); at the same time, low phototoxicity was observed in normal cutaneous keratinocyte HaCaT cells after PDT treatment [162].

These results promote liposomal curcumin as a potential natural photosensitizer that can improve its absorption, safety and efficacy in photodynamic therapy in human cancers.

Table 2 highlights some of the effects of PDT and curcumin nanoformulations in skin cancers, by significantly improving the bioavailability and stability of nanocurcumin, increasing cytotoxicity on malignant cells, but with low phototoxicity on normal keratinocytes, the apoptotic effects and molecular mechanisms by generating high levels of ROS, along with the significant depolarization of mitochondrial membranes, while other in vitro experiments on mice demonstrated significant cell migration in the denuded area and highlighted the modulatory role of nanocurcumin on excessive production and the accumulation of ROS generated by laser irradiation [145,146,155,159,160,162].

### 4.4. Gastrointestinal Cancers

Incidence of gastrointestinal cancer varies over time, from short intervals of 8 years to longer intervals of 20 years, suggesting that it is under the influence of a predestined epigenetic program. It is assumed that, until the onset of cancer, there are several stages that include accumulations of precursor events, which makes the incidence of cancer higher after the age of 40 years [163].

In the natural process of human evolution from youth to old age, inherited native stem cells age and are replaced by new stem cells that are phenotypically labile and prone to turn into cancer cells. New, unstable stem cells can degenerate malign in a short time through a process of methylation in the so-called early exponential phase of accelerated carcinogenesis. In the colon, stem cells in glandular structures are replaced every 8 years [164,165,166].

Colorectal cancer has a very high incidence worldwide, ranking third and second in terms of cancer mortality. The incidence varies a lot depending on the level of socio-economic development, geographical regions, food, cultural level, etc. The incidence is lower in the countries of Africa and South-Central Asia and much higher in Europe, Australia, and North America [167].

By expanding and deepening knowledge about the molecular biology and pathogenesis of cancer, the treatment of various tumors has greatly improved today. However, chemoresistance, recurrence rate and mortality are still far from being resolved. Therefore, ways of prevention, and especially new means of treatment with increased efficiency, are constantly being sought. Treatment for colon, gastric and many other cancers usually involves surgery to remove pathological tissue and locoregional lymph nodes, followed by radiotherapy and chemotherapy with conventional drugs and/or immunotherapy.

Vetha et al., in an in vitro experimental study on a CT26 murine colorectal carcinoma cell line, used 450 nm blue light diode-induced photodynamic therapy (BLED-PDT) at a power of 2.4 mW/cm^2^ for 30 min (6.3 J/cm^2^), together with F127-CUR micelles.

The authors report that cell line carcinoma treated with F127-CUR micelles together with BLED significantly reduced cell density and anticipate that this treatment may be promising for cancer eradication [168].

Şueki et al. have tested curcumin, which is a non-toxic compound that has antitumor properties, to check if it can increase the effectiveness of PDT on resistant cancer cells. For this purpose, PC-3 cancer line of prostate cancer cells and the Caco-2 cell lines of colon cancer were used, which were previously identified as non-resistant and resistant to PDT, respectively. Finally, the authors reported that 5-aminolevulinic acid (5-ALA) -mediated PDT, combined with curcumin, has improved the antitumor efficacy of PDT on cells Caco-2, which is considered a highly resistant cancer cell line [169].

In this scenario, the provision of new drugs and scientific treatment methods based on natural products is part of the research conducted by the group led by de Freitas et al., who studied in vitro effects on Caco-2 intestinal cancer cells treated with curcumin-conjugated silver nanoparticles (CUR-AgNPs) and PDT. The results of the study showed that PDT, in the presence of CUR incorporated into hydrogels consisting of CHT and CS, natural biopolymers, capable of the controlled release of CUR-AgNPs, led to the inhibition of human Caco-2 colon cancer cells [170].

Another attempt to obtain a superior photosensitizer in cancer cell therapy was performed by Tsai et al., which encapsulated curcumin, by crosslinking with chitosan, tripolyphosphate (TPP) and conjugation with epidermal growth factor to target the epidermal growth factor receptor (EGFR), overexpressed on cancer cells. The research targeted two cell lines that were used in this study, including a human gastric cancer cell line (MKN45) and the human gastric (non-cancerous) epithelial mucosa (GHG) cell line, which were irradiated with a light-emitting blue diode (460 nm, 5 ± 0.1 mW, measured on the sample surface) for 30 min, at a dose of 9 J/cm^2^. Curcumin-encapsulated chitosan/TPP nanoparticles showed a superior PDT effect in the cancer cells, with a four-fold decrease in IC50. This mode of therapy is promising against cancers that overexpress EGFR [171].

Human hepatoblastoma (HB) is the most common form of liver cancer in infants and children under 5 years of age [172].

To reduce the tumor, but especially to eradicate circulating tumor cells, chemotherapy is used before and after surgery. Despite all the positive results, some cases with extensive or recurrent tumors are a major problem, due to the emergence of drug resistance. Ellerkamp et al. investigated in vitro hepatoblastoma cell lines (HuH6, HepT1) and hepatocellular carcinoma cell lines (HepG2, HC-AFW1) treated with curcumin and exposed to blue light (480 nm, 300 W, for 10 s), and revealed decreased cell viability and a significantly increased level of ROS [173].

The emergence of multidrug resistance (MDR) is a major impediment to the long-term success of therapy against various cancers. It is already known that P-glycoprotein (P-gp) is a membrane transporter, which is ATP-dependent, and it has the function to drain the drug molecules from the cancer cell, so that chemotherapy is less effective. In their adaptive evolution, cancer cells protect themselves by increasing P-gp expression, thus avoiding chemotherapy-induced cellular degradation [174,175].

PDT has become an attractive method of treatment for hepatocellular carcinoma, because it is easy to administer and does not affect normal tissues.

To avoid the effects of chemoresistance, Li et al. have developed a new ICG & Cur @ MoS2 system (ICG and Cur represent indocyanine green and curcumin respectively) nanoplatform, which can perform photothermal-photodynamic therapy and inhibit P-gp efficiently and safely. The researchers used HepG-2 cells (human hepatoma cells) cultured in vitro with ICG and Cur @ MoS2 and irradiated with an 808 nm NIR laser at 2.0 W/cm^2^ for 5 min to evaluate the photothermal effect. Acute toxicity was investigated in vivo in female mice, in which the tumor was irradiated with an 808 nm (1.2 W/cm^2^) NIR laser for 5 min. Cell apoptosis was significant in the ICG @ MoS2 and NIR group, indicating that it was induced by heat and ROS. MRNA decreased significantly in the ICG and Cur @ MoS2 group, indicating that ICG and Cur @ MoS2 inhibited MDR1 transcription. In conclusion, the ICG and Cur @ MoS2 nanoparticles, under the action of PTT-PDT, have inhibited P-gp effectively, and may have great potential in the treatment of hepatocellular carcinoma [176].

### 4.5. Lung Cancer

Globally, lung cancer is one of the leading causes of death in both men and women. The small cell form represents about 80% of all types of lung cancer, and the one through which deaths are more common, because the diagnosis is generally made when the disease is quite advanced. After the approval of modern biological therapy, the number of deaths was substantially reduced. However, there are special problems because the disease continues to progress in most cases, due to the development of drug resistance [177,178,179].

As in other forms of cancer due to these problems, research continues to discover drugs or alternative ways to overcome the phenomenon of drug resistance.

Jiang et al. investigated the effects of the new Cur-SLN photosensitizer on A549 cells of small lung cancer cells irradiated with a 430 nm light-emitting diode (LED; power density 50 mW/cm^2^) for 20 min. Cur-SLNs were obtained by emulsification and solidification at low temperature. The organic phase with 0.1 g lecithin, 0.15 g Cur and 0.2 g stearic acid was dissolved in 10 mL of chloroform. Moreover, 0.2 g of polyoxyethylene stearate (40) (Myrj52) dissolved in 30 mL of deionized water formed the aqueous phase. The organic phase was injected into the aqueous phase and stirred until the organic solvent disappeared. Then, in an environment at 0–2 °C, 10 mL of cold water were added and stirred at 1200 rpm. The supernatant was removed by centrifugation and the precipitate was washed twice with deionized water, resuspended in ultrapure water, and refrigerated at −80 °C, and finally lyophilized. The results revealed that Cur-SLN increased the expression of caspase-3, caspase-9, and promoted the Bax/Bcl-2 ratio, which demonstrated that this new Cur photosensitizer significantly induced apoptosis in A549 cells for this type of lung cancer [180].

In the last decade, the delivery agents of photosensitizing nano-carriers have been developed a lot, and those who respond very well to near-infrared (NIR) lasers have received special attention because they absorb light very strongly from 700–900 nm.

Local temperature released from the interaction of NIR light with the photosensitizer deeply destroys cancer cells, which gave high hopes for the beneficial effect of eradicating tumors; however, the damage to nearby tissues is still an unresolved issue.

Its outstanding optical properties and biocompatibility have helped Indocyanine Green (ICG) to be FDA approved as a photosensitizer for NIR in clinical use [25,181,182].

Huang et al. developed an antitumor product consisting of a green photosensitizer with indocyanine (ICG) that was co-encapsulated with curcumin (CUR) in liposomes (LPs), followed by conjugation of the GE11 peptide to provide targeting effects to cancer cells that express on their surface epidermal growth factor receptor (EGFR). A fresh medium containing CUR/ICG, free CUR/ICG-LPs and GE11-CUR/ICG-LPs was added to A549 non-small lung cancer cells line, HeLa human cervical cancer cells and LO2 human normal liver cells, incubated for 24 h, followed by 808 nm NIR laser irradiation, (1W/cm^2^) for 0, 5, and 10 min. In this study, after the administration of PDT, the ICG photosensitizer generated an increase in temperature in the tumor area and, on the other hand, the GE11-CUR/ICG-LPs complex slowly released CUR, obtaining strong anticancer effects. The research results showed that GE11-CUR/ICG-LPs, together with PDT, could induce the apoptosis of cancer cells by promoting ROS generation and cell cytoskeleton disruption by the increased stimulation of apoptotic signaling pathways and the inhibition of the EGFR-mediated PI3K/AKT pathway [183].

Another research study to obtain a photosensitizer that is easier to administer and with more effective properties was completed by Baghdan et al. They obtained curcumin-loaded PLGA nanoparticles (PLGA.CUR.NPs) as nano-in-microparticles for inhalation administration. In vitro irradiation results on human lung epithelial carcinoma cells (A549) showed a significant increase in cellular phototoxicity, but dependent on the radiation dose used by the 457 nm LED device. In short, the authors argue that nano-in-microparticles are promising carriers of drugs for the photodynamic therapy of lung cancer [184].

### 4.6. Other Cancers

Glioblastoma multiforme accounts for approximately 80% of primary malignant brain tumors. Despite current innovations in cancer treatment, this type of tumor has a disappointing prognosis [185].

From the results of several randomized international studies reported so far, it can be seen that they have failed to achieve convincing results with long-term survival after immunotherapy [186,187].

Because there is no reasonable treatment in glioblastoma multiforme, the use of PDT with different CUR nanoparticles is being tested experimentally in vitro. In a recent research study, Kielbik et al. analyzed the cytotoxic effects of CUR alone and as a photosensitizer on glioblastoma SNB-19 cells. After incubation with CUR and irradiation with blue light (6 J/cm^2^), over 90% of glioblastoma SNB-19 cells underwent apoptosis [188].

Jamali et al. used biodegradable polymeric nanoparticles (PLGA NPs) conjugated with the anti-EGFRvIII monoclonal antibody (MAb-CUR-PLGA NPs) to enhance the photodynamic action of CUR on overexpressed EGFRvIII cells (DKMG/EGFRvIII cells) of glioblastoma tumors. The results demonstrated in vitro that following PDT, the photocytotoxicity of MAb-CUR-PLGA NPs was significantly higher than that of CUR-PLGA NPs in the DKMG/EGFRvIII glioma cells. The authors propose that anti-EGFRvIII MAb-CUR-PLGA NPs should be used as a PDT-specific photosensitizer in overexpressed EGFRvIII tumor cells [189].

Curcumin has been used as a PS in PDT and has been the subject of numerous studies, due to its valuable antiviral, antimicrobial, and especially antineoplastic effects in various forms of human cancer [190,191,192].

Kazantzis et al. investigated the natural characteristics of bisdemethoxy curcumin regarding their efficacy together with PDT in vitro on prostate cancer cells. In these experiments, all four curcuminoids released enough ROS that produced cytotoxicity after PDT, with a significant reduction in prostate cancer LNCaP cells [193].

## 5. Final Remarks and Conclusions

**Figure 4:**
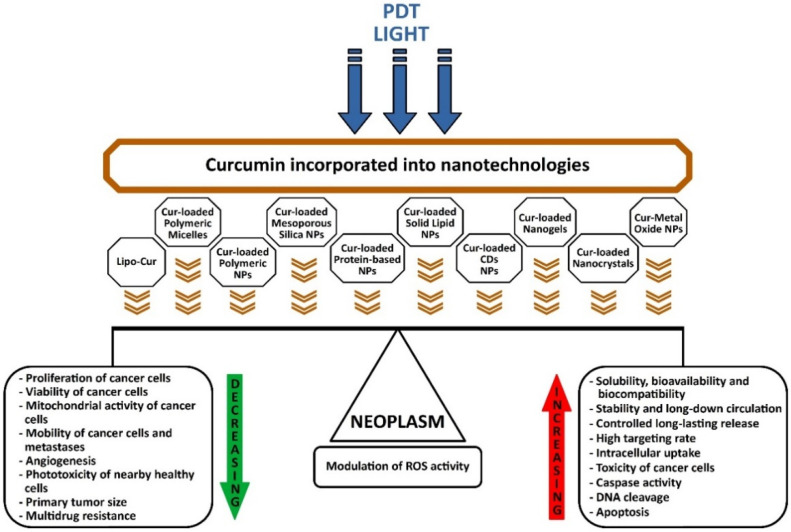
Effects of latest innovations and nanotechnologies with curcumin applied in the photodynamic therapy (PDT) of cancer.

As revealed by all the above-mentioned revised studies, the latest nanoformulations of curcumin applied in PDT illustrate its complex actions on tumor cells, as shown in Figure 4.

Although curcumin is a polyphenol that has been used in Ayurvedic medicine for many years, as well as a spice for food fragrance, food coloring or adjuvant, its pharmacological complex, anti-inflammatory, antiviral, anti-bacterial, antifungal, anti-neurodegenerative, and its anti-cancer properties have been intensively investigated in recent years. Therapeutic applications of curcumin have been limited, due to its extremely low solubility, instability in body fluids and rapid metabolism. Nanomedicine and the latest nanotechnologies have shown excellent potential to improve the solubility, biocompatibility and therapeutic effects of curcumin.

State-of-the-art research presented here in the photodynamic therapy of various forms of cancer has been able to highlight the significant anticancer potential of curcumin as a valuable photosensitizer when it is combined with nanotechnologies.

In all in vitro or in vivo experiments performed for the study of curcumin nanoformulations, it has been shown to have a high encapsulation efficiency and long-term controlled release, increasing its solubility, bioavailability and biocompatibility, as well as stability and long-down circulation.

Curcumin incorporated into nanotechnologies has a higher intracellular absorption, a superior targeting rate, increased toxicity to cancer cells, accelerates the activity of caspases and DNA cleavage, and ultimately induces apoptosis.

PDT applied together with nanoformulations of curcumin, diminishes the mitochondrial activity of cancer cells, decreases the viability and proliferation of cancer cells, drops the angiogenesis, the cancer cells’ mobility and metastases.

The experiments included in this review showed that PDT and nano-curcumin reduce the size of the primary tumor and invert the multidrug resistance in chemotherapy and decrease the resistance to radiotherapy in neoplasms.

PDT applied with curcumin nanoformulations has been shown to have no significant phototoxic effects on healthy cells near the treated tumor.

Regarding breast cancer that came first in terms of incidence, recent experimental research has shown that nanoformulations of curcumin together with PDT can contribute to its treatment by deep infiltration into cancer cells, high cytotoxic action, shrinking tumor volume and stopping metastases.

Current research has shown that the use of PDT and curcumin nanoformulations have a modulating effect on ROS generation, so light or laser irradiation will lead to excessive ROS growth, while nano-curcumin will reduce the activation of ROS-producing enzymes, or will determine the elimination of ROS, seemingly opposite but synergistic phenomena by inducing neoplasm apoptosis, but at the same time, accelerating nearby tissue repair.

All studies reviewed and presented revealed the great potential for applicability of PDT and curcumin nanoformulations in cancer therapy.

This review paves the way for further investigations and advances in this field.

## Figures and Tables

**Table 1:** Curcumin nanoformulations and PDT in breast cancer.

References	Type of Study	Type of Light and Curcumin	Total Energy (J) Applied	Analyzed Parameters	Conclusions
[94]	In vitro experimental 4T1 mouse breast cancer cells.	Blue light 450 nm. Curcumin nanodrugs (Cur NDs)	Blue light (P = 640 mW) with a wavelength of 450 nm at a fixed distance of 13 cm for 1 min.	In vitro cytotoxicity. Intracellular ROS production was analyzed using an intracellular ROS kit. c-Jun N-terminal kinase (JNK); p-JNK; mitogen-activated protein kinase (MAPK); caspase-3 (Casp 3); Bcl-2–associated X (Bax); Glyceraldehyde 3-phosphate dehydrogenase (GAPDH).	This study proved that Cur NDs could be a full of promise PS for speeding up the performance and reliability of PDT against breast cancer, with very good prospects for use in clinical practice.
[98]	In vitro breast cancer model, MCF-7 cells.	LED 440 (±10) nm, with 420 mW output power, 2.52 W total power, with 209 W/cm^2^ irradiance. Curcumin-nanoemulsion (CNE).	80 J/cm2 fluency, set at 6.4 s/application	Caspases 3 and 7 activity. Estimation of intracellular reactive oxygen species production by 2′-7′-Dichlorodihydrofluorescein diacetate (DCFH-DA) technique.	Curcumin-nanoemulsion and PDT increased the activity of capsases 3 and 7, had a phototoxic effect with a significant reduction in MCF-7 cell proliferation and stimulated ROS release; this association has great prospects for breast cancer therapy.
[102]	In vitro experimental model of circulating tumor cells (CTCs) with human breast cancer cells (MDA-MB-231, ATCC HTB-26).	Blue light (447 nm, 100 mW). CUR-NPs.	MDA-MB-231 cells were laser irradiated for 30 min under flow conditions (5 cm s^−1^).	Cell viability assay Morphology of nanoparticles Photodynamic inactivation Scanning electron microscopy of circulating breast cancer cells. CLSM micrographs showing cellular curcumin accumulation and photodynamic effect of curcumin loaded nanoparticles (CUR-NPs) on circulating MDA-MB-231 cells.	Apoptosis and necrosis of metastatic malignant cells were demonstrated by this experimental study in vitro on human breast cancer cells, using CUR-PLGA NPs and 30 min laser irradiation (447 nm and 100 mW) under continuous flow conditions. Results open new perspectives in clinical oncology for targeting metastases.
[103]	In vitro MCF-7 Human Breast Cancer Cell-Line.	LED 430-nm GaAlAs, CW. In vitro release of Curcumin Nanostructured Lipid Carriers (CUR-NLCs) formulas.	Irradiation protocol: blue light (430 nm) for 5 min (power 100 mW), spot size radius 4 cm, irradiance 2 mW/cm^2^, fluence 6 J/cm^2^).	Determination of encapsulation efficiency and drug loading percentages by spectrophotometry measurements. Morphology changes by transmission electron microscopy. Dark and photo-cytotoxicity studies of MCF-7 cells survival.	Carriers of nanostructured lipids loaded with curcumin and olive oil used in conjunction with PDT have increased the potency of penetrating breast cancer cells and the cytotoxic activity. The results of the study suggest that CUR-NLCs in low doses after exposure to blue light have a significant anticancer effect in breast cancer.
[109]	In vivo on the breast cancer model in Female Balb/c mice (6 to 8 weeks).	Blue diode laser at 450nm, CW for PDT, and 808 nm in NIR range for PTT. An external magnetic field was applied for appropriate delivery of the drug. Curcumin on silica-coated Fe_3_O_4_ nanoparticles.	In vivo experiment: with female Balb/c tumorized mice, divided into 6 groups: (I) PBS injection (control group). (II) Curcumin plus irradiation with a blue diode laser at 450 nm with 150 mW/cm^2^ for 3 min (CUR + PDT group). (III) Blue diode laser for 3 min followed by NIR laser with 0.5W/cm^2^ for 7 min (Blue + NIR lasers group). (IV) injection of 40 µL NC (NC group). (V) injection of 40 µL of NC solution containing 20 µg curcumin (0.46 mg/mL) plus irradiation with NIR laser at 808 nm for 7 min (NC + PTT). (VI) injection of 40 µL of NC containing 20 µg curcumin plus irradiations with two lasers with up-mentioned intensities and exposure times, while a rigid magnet was fixed on the tumor to maintain the injected NC in the tumor position (NC + PDT + PTT group).	Analysis of expression of apoptotic proteins Bax and Caspase 3. In vitro toxicity of Fe_3_O_4_/ SiO_2_ NPs after 24 and 48 h. In vitro release of curcumin from the NCs. Antitumor effect of nanocomposite plus PDT and PTT approach in vivo.	In the group treated with NC+PDT+PTT the tumor volume was significantly reduced and the expression of proapoptotic proteins Bax and Caspase 3 increased significantly compared to the control group, without weight loss, no adverse effects on vital organs in the mice autopsy images. Method could replace chemotherapy for triple-negative breast cancers.
[110]	In vitro study of human breast adenocarcinoma MCF-7 cells.	5 min irradiation at 450 nm and 100 mW/cm^2^. Ga(III)-curcumin complex.	6 mW/cm^2^ and the exposure times were 167 s, 334 s, 501 s, 1002 s, and 6012 s yielding 1, 2, 3, 6 and 10 J/cm^2^ fluence, respectively.	Photophysical and photochemical studies (UV-Visible absorption and fluorescence; ROS production; in vitro cytotoxicity assay. Dark cytotoxicity.	Administration of the Ga (III) -curcumin complex studied on MCF-7 breast cancer cells has shown that metal complexation increases its photodynamic effect compared to simple curcumin.

**Table 2:** Nanocurcumin in skin cancer therapy.

References	Type of Study	Type of Light and Curcumin	Total Energy (J) Applied	Analyzed Parameters	Conclusions
[145]	In vitro A431 -human cell line model (epidermoid carcinoma cell line) and HaCaT cells (spontaneously transformed aneuploid immortal keratinocyte cell line from adult human skin)	Ultraviolet radiation B (UVB) Demethoxycurcumin (DMC)	UVB (10–100 mJ/cm^2^)	Inhibition of tumor cell growth. Enhancement of apoptosis in cells. Apoptosis-associated proteins including Bcl-2, Mcl-1, Bax, nuclear factor-κB (p65), p-p65, p53, caspase-3, caspase-9, and cytochrome C. Measurement of ROS (which increased significantly). Analysis of mitochondrial potential (which decreased: important depolarization occurred).	PDT by ultraviolet B radiation and DMC have experimentally succeeded in causing apoptosis in skin cancer cells. DMC may be a promising photosensitizer for PDT to eradicate skin cancer cells.
[146]	*In-vitro/In-vivo* studies and histopathological examination on a human skin cancer cell line (A431)	Blue light (410 nm); PEGylated lipid nanocarriers (PLN) loaded with curcumin (Cur).	In vitro 300 mW/cm^2^ for 4 min by blue light. In vivo LED (420 nm) for 10 min at a fluence of 90 mW/cm^2^	Fluorescence intensity measured by confocal laser microscopy. Histopathological studies. In-vitro cytotoxicity.	This in vitro study with Cur-loaded PLN together with blue light proved a significantly higher cytotoxicity than the control sample against human epidermoid squamous cell carcinoma cell line (A431). In vivo study showed a significant improvement in skin carcinoma after photodynamic therapy and Cur-loaded PEGylated lipid nanoparticles. Beneficial effects of this safe and economical method, bring hope in the treatment of cancer.
[155]	In vitro experiments on mouse embryonic fibroblasts (MEFs) cells	Diode laser device with a wavelength of 450 nm and an output power of 75 mW. Dendrosomal Nano-Curcumin (DNC)	Cells were irradiated for 224 s (for getting a dose of 17.9 J, with an energy density of 0.63 J/cm^2^), and 337 s (for getting a dose of 26.9 J, with an energy density of 0.95 J/cm^2^). For other doses, the time was set in the same way.	- RNA extraction was quantified by spectrophotometry - cDNA synthesis - TGF-β, VEGF, TNF-α, IL-6 and glyceraldehydes 3-phosphate dehydrogenase (GAPDH). In vitro migration assay for cell motility; cell cycle analysis by flow cytometry; quantitation of DNA content stained. Measurements of intracellular ROS.	Results revealed a notable proliferation of mouse embryonic fibroblasts after the combination of DNC + LLLT (450 nm) at a dose of 0.95 J/cm^2^. Simultaneous exposure to DNC +LLLT enriched S-phase entry and increased proliferation as well as significant migration of MEF cells in the denuded area, up-regulating growth factors (TGF-β, VEGF) and shortening the inflammatory phase by modulating cytokines (TNF-α, IL-6). Combined therapy (DNC + LLLT) also highlights the modulating role of nanocurcumin on the production and excessive accumulation of ROS generated by laser action.
[159]	In vitro studies on curcumin + PDT on melanotic (A375) and amelanotic melanoma (C32) cell lines.	Lamp with polarized light with power density set to 20 mW/cm^2^, blue light (380–500 nm), including maximum absorption of curcumin (410 nm). Curcumin dissolved in dimethyl sulfoxide (DMSO).	Irradiation time = 5 min (6 J/cm^2^).	MTT cell viability assay. Cell death evaluation by neutral comet assay (NCA). Fluorescent staining of actin filaments Caspase-3 immunocytochemical staining. Holotomographic microscopy studies. Cell viability and phototoxicity.	PDT + curcumin increased the number of apoptotic and necrotic cells compared to the control without irradiation, it induced overexpression of caspase-3 and DNA cleavage and low cell proliferation due to reorganization of the actin cytoskeleton. PDT together with curcumin can be an effective way to induce apoptosis in melanoma.
[160]	Experimental study on malignant melanoma C540 (B16/F10) cell line.	808-nm laser. Ultrasound (US). Nanoparticles of selenium- polyethylene glycol-curcumin (Se-PEG-Cur).	Output power = 1000 mW Power density = 1.0 W/cm^2^. Irradiation time = 10 min. US output power of 1.0 W/cm^2^; Frequency of 1MHz; Irradiation time = 1 min.	Detection of intracellular ROS Viability of C540 (B16/F10) cells. Fluorescence intensity (FI).	Se-PEG-Cur can be a very good photosensitizer for phototherapy plus sonotherapy in the destruction of melanoma cancer cells through thermal and ROS-generating effects.
[162]	Experiments on melanoma (MugMel2), squamous cell carcinoma (SCC-25), and normal human keratinocytes (HaCaT) cell lines.	Blue light (380–500 nm); 20 mW/cm^2^. Liposomal Curcumin.	Irradiation time = 2 min; 2.5 J/cm^2^	Impact of Liposomal Curcumin on Cells Lines’ Apoptosis. Bax and Bcl-2 Expression. Cell Viability Assay. Wound-Healing Assay.	Experimental study demonstrated a significant improvement in the bioavailability and stability of liposomal encapsulated curcumin as a potent apoptotic photosensitizer in squamous cell carcinoma (SCC-25) and melanoma (MugMel2). Low phototoxicity was observed in normal cutaneous keratinocyte HaCaT cells after PDT treatment.

## Data Availability

The references used to support the findings of this study are available from the first (L.M.A.) and the corresponding (G.L.) authors upon request.

## Abbreviations

5-Aminolevulinic acid	(5-ALA)
Activated partial thromboplastin time tests	(aPTT)
Adenosine triphosphate	(ATP)
Basal cell carcinoma	(BCC)
Baseline or ground state	(S0)
B-cell lymphoma 2	(Bcl-2)
Bcl-2–associated X	(Bax)
Blue light-emitting diode	(BLED)
Blue-light-emitting diode- photodynamic therapy	(BLED-PDT).
Cervical intraepithelial neoplasia	(CIN)
Chitosan	(CHT)
Cholesteryl hemisuccinate	(CHEMS)
Chondroitin sulfate	(CS)
Circulating tumor cells	(CTCs)
Colorectal cancer	(CRC)
Confocal laser scanning microscopy	(CLSM)
Continuous wave	(CW)
Curcumin	(Cur)
Curcumin conjugated silver nanoparticles	(CUR-AgNPs)
Curcumin loaded PLGA nanoparticles	(CUR-PLGA NPs)
Curcumin nanocrystals	(Cur-NCs)
Curcumin nanoparticles	(CUR-NPs)
Curcumin-loaded liposomes	(Lipo-Cur)
Curcumin-nanoemulsion	(CNE)
Curcumin-loaded solid lipid nanoparticles	(Cur-SLNs)
Cutaneous squamous cell carcinoma	(cSCC)
Cyclodextrins	(CDs)
Demethoxycurcumin	(DMC)
Dendrosomal Nano Curcumin	(DNC)
Deoxyadenosine triphosphate	(dATP)
Dichlorodihydrofluorescein diacetate	(DCFH-DA)
Dioleoyl phosphatidylethanolamine	(DOPE)
Dissolved in dimethyl sulfoxide	(DMSO)
Distearoyl phosphoethanolamine	(DSPE)
EGFRvIII overexpressed human glioblastoma cell line	(DKMG/EGFRvIII cells)
Epidermal growth factor	(EGF)
Epidermal growth factor receptor	(EGFR)
European Medicines Agency	(EMA)
Ferric chloride hexahydrate	(FeCl3·6H2O)
Ferrous sulfate heptahydrate	(FeSO4·7H2O)
Fluorescein isothiocyanate	(FITC)
Fluorescence microscopy imaging system	(FMI)
Food and Drug Administration	(FDA)
Fourier-transform infrared spectroscopy	(FTIR)
Glyceraldehydes 3-phosphate dehydrogenase	(GAPDH)
Immunohistochemistry	(IHC)
Indocyanine Green photosensitizer	(ICG)
Laser irradiation in near-infrared	(NIR)
Light emitting diode	(LED)
Liposome nanocarriers curcumin	(LIP-CUR)
Liposomes	(LPs)
Mesoporous silica nanoparticles	(MSNs)
Monoclonal antibody	(MAb)
Monoclonal antibody against EGFRvIII	(A-EGFRvIII-f)
Mouse embryonic fibroblasts	(MEFs)
Multidrug resistance	(MDR)
Multidrug resistance protein 1	(MDR1)
Myeloid cell leukemia 1	(Mcl-1)
Nanocomposite	(NC)
Nanoparticles	(NPs)
Nanoparticle	(NP)
Nanostructured lipid carriers	(NLCs)
Neutral comet assay	(NCA)
Notch receptor blocker human	(DAPT)
Nuclear Factor-Kappa-B	(NF-κB)
Papillomavirus	(HPV)
Polyethylene glycol	(PEG)
PEGylated lipid nanocarriers	(PLN)
P-glycoprotein	(P-gp)
Phosphate buffer saline	(PBS)
Photodynamic inactivation	(PDI)
Photodynamic therapy	(PDT)
Photosensitizer	(PS)
Photothermal therapy	(PTT)
Poly (lactic-co-glycolic acid) nanoparticles	(PLGA NPs)
Poly (ethylene glycol)	(PEG)
Poly (lactic acid)	(PLA)
Poly (lactic-co-glycolic acid)	(PLGA)
Poly (ε-caprolactone)	(PCL)
Poly (ethylene glycol) polymer	(PEG)
Polyoxyethylene(40)stearate	(Myrj52)
Polyvinylpyrrolidone	(PVP)
Pressure ulcers	(PU)
Reactive oxygen species	(ROS)
Scanning electron microscopy	(SEM)
Silver nanoparticles	(AgNPs)
Excited Singlet state	(S1)
Solid lipid nanoparticles	(SLNs)
Squamous cell carcinoma	(SCC)
Tetraethyl orthosilicate	(TEOS)
The half maximal inhibitory concentration	(IC50)
Transforming growth factor beta	(TGF-β)
Tripolyphosphate	(TPP).
Tumor necrosis factor alpha	(TNF-α)
Tumor Nodes Metastasized	(TNM)
Ultraviolet A	(UVA)
Ultraviolet radiation B	(UVB)
Vascular endothelial growth factor	(VEGF)
Viability measurements	(MTT)
Zinc oxide nanoparticles	(ZnONP_CS_)
